# Simple Model for Corrugation in Surface Alloys Based on First-Principles Calculations

**DOI:** 10.3390/ma13194444

**Published:** 2020-10-07

**Authors:** Monika Nur, Naoya Yamaguchi, Fumiyuki Ishii

**Affiliations:** 1 Graduate School of Natural Science and Technology, Kanazawa University, Kakuma, Kanazawa 920-1192, Japan; monika@cphys.s.kanazawa-u.ac.jp; 2 Senior High School 8 Padang (SMAN 8 Padang), Education Department of West Sumatera (Dinas Pendidikan Provinsi Sumatera Barat), Padang 25179, Indonesia; 3 Nanomaterials Research Institute, Kanazawa University, Kakuma, Kanazawa 920-1192, Japan; n-yamaguchi@cphys.s.kanazawa-u.ac.jp

**Keywords:** first-principles calculation, surface alloys, corrugation, atomic radii

## Abstract

The structural stability of *M*/Ag(111)–$\left(\sqrt{3}\times \sqrt{3}\right)R30{}^{\circ}\;$ surface alloys is systematically investigated by using first-principles calculations, where *M* is a member of group III (B, Al, Ga, In, Tl), IV (C, Si, Ge, Sn, Pb), and V (N, P, As, Sb, Bi) elements. We focus on the corrugation parameter *d* which is determined by the height of the *M* atom from the Ag atom in the plane of the top-most atom, and the relation between atomic radii and corrugations in *M*/Ag(111) is obtained. The tendencies of the corrugation parameter *d* can be understood by using a simple hard spherical atomic model. We introduce a new type of atomic radii determined by the corrugation in surface alloys, *surface alloy atomic radii*, which can be useful for rapid predictions of the structures of surface alloys, not only for *M*/Ag (111)–$\left(\sqrt{3}\times \sqrt{3}\right)R30{}^{\circ}$ systems but also for other surface alloys.

## 1. Introduction

Surface alloys are a combination of a metallic substrate and atoms of different elements; that is, a few layers of surface atoms in substrate are alloyed with added atoms. A large number of possibilities of combining the substrate and added atoms allow flexibility in the electronic properties [1], which can be related to many varied applications such as spintronics [2], catalysts [3], two-dimensional superconductors [4], and corrosion and passivation [5]. In this context, different types of surface alloys have been widely investigated [6,7,8,9,10,11,12,13] and to elucidate the structural properties of structure alloys has been a crucial step to understanding the origin of such a wide range of phenomena.

When new atoms are added to the substrate, the height of the added atoms is generally different from that of the atoms at the surface of the substrate, due to mismatches of the atomic size between the added and substrate atoms. This difference is known as a corrugation parameter, and it is one of the key quantities that characterizes surface alloys. There have been many studies regarding the corrugation parameters and the values of the corrugation parameters depend on a combination of a surface system and additional atoms. Understanding and controling the corrugation parameters are very important research topics. As an example, breaking the inversion symmetry at the surface leads to Rashba momentum splitting, which is an important property for spintronic applications. It is known that the size of Rashba momentum splitting depends on the corrugation parameter [14].

As the corrugation parameter of a surface alloy is inherently related to atomic sizes [6], the concept of an atomic radius becomes a crucial factor in determining the parameter.

There are many types of atomic radii. Which type is effective depends on chemical bonding in materials, such as the covalent radius, metallic radius, and Van der Waals radius. It is unclear which atomic radius is suitable for predicting corrugation in surface alloys. By investigating the corrugation parameters on the surface alloys as a simple system, we can provide a new type of atomic radii for surface alloys, which can be utilized to predict corrugation in other complex surface alloys.

In this study, we focused on silver surface alloy systems and explored the *M*/Ag (111) surface alloys, where *M* is of group III (B, Al, Ga, In, Tl), IV (C, Si, Ge, Sn, Pb), and V (N, P, As, Sb, Bi). The systematic calculations are performed for simple $\left(\sqrt{3}\times \sqrt{3}\right)R30{}^{\circ}$ structures widely observed in experiments, which are formed on a (111) surface plane of a face-centered-cubic lattice [7,8,9,10]. The simple hard spherical atomic model was used to obtain corrugation parameter *d* defined by the height of the *M* atoms from the Ag atoms in the top atomic plane. Furthermore, the concept of atomic radii in surface alloys is introduced to determine the corrugation parameter. Our finding leads to predictions of surface alloy structure of not only *M*/Ag(111)–$\;\left(\sqrt{3}\times \sqrt{3}\right)R30{}^{\circ}$, but also other complex surface alloys.

## 2. Materials and Methods

We performed density functional calculations within the local spin density approximation [15,16] using OpenMX code [17]. The norm-conserving pseudopotentials are adopted [18] with an energy cut off of 200 E_0_, where E_0_ is 13.605 eV, and used 8 × 8 × 1 k-point mesh. The numerical pseudo atomic orbitals [19] were utilized as follows: For most models, the numbers of the *s*-, *p*-, and *d*-character orbitals were three, two, and two, respectively—especially three, three, and two for Pb, Sb, and Bi. The cut off radii of Ag, B, Al, Ga, In, Tl, C, Si, Ge, Sn, Pb, N, P, As, Sb, and Bi were 7.0, 7.0, 7.0, 7.0, 7.0, 8.0, 5.0, 6.0, 7.0, 7.0, 8.0, 5.0, 7.0, 7.0, 7.0, and 8.0, respectively, in units of a_0_, where a_0_ is the Bohr radius, 0.529177 Å. These choices of parameters for pseudo atomic orbitals are well tested for bulk systems [17].

**Figure 1:**
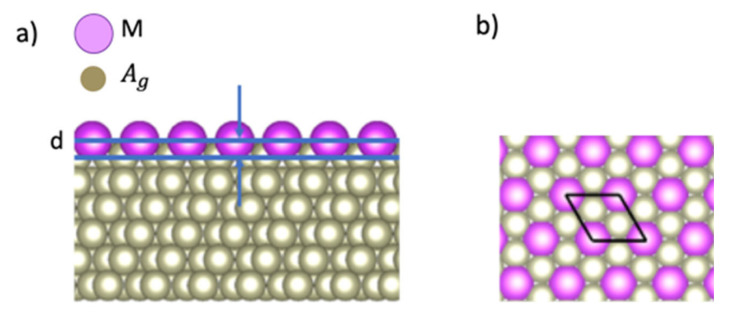
Atomic structure of an *M*/Ag(111)–$\left(\sqrt{3}\times \sqrt{3}\right)R30{}^{\circ}$ surface alloy. (**a**) Side view. *d* denotes the corrugation parameter; (**b**) Top view. The rhombus represents the unit cell. The in-plane cell length is $\left(\sqrt{3}\right)$*a*_Ag_, where *a*_Ag_ is the lattice constant for Ag(111).

Figure 1 shows a computational model of a surface alloy composed of *M* atoms and a (111) surface of a face-centered-cubic Ag crystal, where 1/3 of Ag atoms on the topmost atomic layer were replaced with *M* atoms. The height of *M* atoms is defined from the Ag atoms in the top atomic plane as the corrugation parameter *d* (see Figure 1a). The 6-atomic-layer supercell model (5 Ag atomic layer + 1 *M*/Ag atomic layer) is adopted with a cell length of 47.34 Å along the direction normal to the Ag(111) plane. Each of the Ag layers has 3 Ag atoms and each *M*/Ag layer has 1 *M* atom and 2 Ag atoms. We used a $\left(\sqrt{3}\times \sqrt{3}\right)R30{}^{\circ}$ surface structure (see Figure 1b) and an experimental value of a lattice constant *a*_Ag_ = 4.1 Å for Ag substrates [20]. Figure 1b also shows the rhombus which represents the surface unit cell. The in-plane cell length is $\sqrt{3}$ *a*_Ag(111)_ (5.021 Å), where *a*_Ag(111)_ is the in-plane lattice constant for an Ag(111) surface, which is given as *a*_Ag_ /$\sqrt{2}$ (2.899 Å). To treat the surface alloys with the surface slab model, the atomic positions are fixed for the bottom three Ag layers and relaxed the atomic positions for the top three layers. The convergence of corrugation parameters is checked for the thickness of slab models up to 10 layers with half of the atomic layers fixed. For example, the difference for the corrugation parameter *d* of Bi/Ag (111) is less than 0.03 Å.

## 3. Results and Discussions

### 3.1. Corrugation Parameter of M/Ag(111)–$\left(\sqrt{3}\times \sqrt{3}\right)R30{}^{\circ}$

Calculated corrugation parameters for 15 *M* elements of the III, IV, and V groups are shown in Table 1. The corrugation is positive when the atomic number Z of *M* atoms is larger than that of Ag atoms (Z = 47). Positive corrugation means that the positions of *M* atoms are over the topmost Ag atomic layer (see Figure 1a). The corrugation is negative for Z smaller than 47. Negative corrugation means that the positions of *M* atoms are under the topmost Ag layer.

Negative corrugation for the atoms of the first row (B, C, and N) and second row(Al, Si, and P) have a tendency that the value of corrugation *d* decreases in the same row of the periodic table in line with the increasing atomic number. However, for Ga, Ge, and As, there is no clear tendency. The values of corrugation which are positive for In, Sn, and Sb tend to decrease by the atomic number increase in the same row of the periodic table; *d* are 0.190, 0.184, and 0.178 Å for Z of 49, 50, and 51, respectively. However, the corrugation of Tl, Pb, and Bi increase if the atomic number increases in the same row of the periodic table; *d* are 0.306, 0.556, and 0.690 Å for Z of 81, 82, and 83, respectively.

There were not so many experimental and theoretical studies for the corrugations in *M*/Ag (111); we only obtained values of corrugation parameters for *M* = Ge, Pb, and Bi [9,14,21,22,23,24]. The calculated corrugations of Pb and Bi are comparable to the experimental results at finite temperature [9,22] and other calculations [9,14,22,23,24], while for *M* = Ge, we obtain a result of −0.05 Å different from the experimental value of 0.3 Å [21].

**Figure 2:**
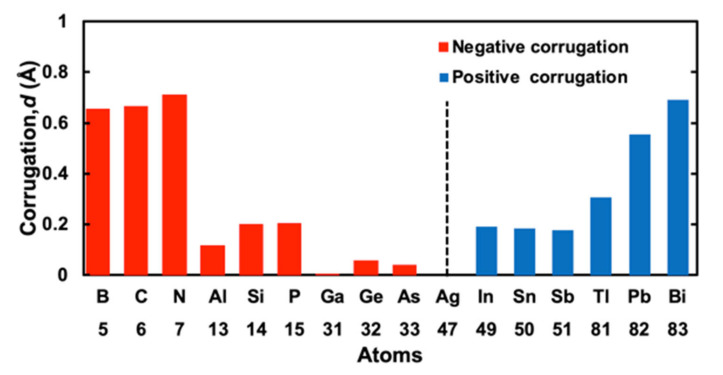
Summary of calculated corrugation parameter *d* of *M* atoms that consist of group III, IV, and V atoms, sorted by atomic number Z. Red and blue bars correspond to negative and positive values, respectively.

In Figure 2, the corrugation values of 15 *M* cases and an Ag case are plotted as absolute values. To distinguish positive corrugation parameters from negative ones, we gave red color for negative corrugation and blue color for positive corrugation. From Figure 2 and Table 1, we can see the tendency of the corrugation parameters which we already discussed above. For negative corrugation, the value of corrugation ranges from −0.002 to −0.712 Å, while for positive corrugation, it ranges from 0.178 to 0.690 Å. There may be a different mechanism between negative and positive corrugations. The differences of the sign of corrugation may depend on mismatches of the atomic size of an *M* atom and that of an Ag atom: positive for a larger *M* atom and negative for a smaller one.

### 3.2. Simple Hard Spherical Atomic Model for Surface Corrugations

To discuss the origin of the differences in corrugation for 15 *M* cases, we constructed a simple hard spherical atomic model (SHSAM). Here, we assumed that atoms were hard spheres with an atom-dependent radius, that is, atomic radius. The surface corrugation can be determined by the atomic radius of *M* atoms and atomic radius of Ag atoms through the SHSAM.

**Figure 3:**
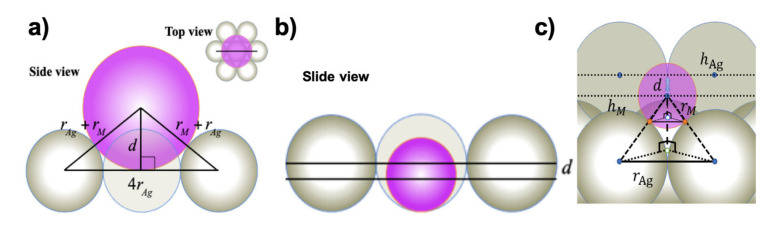
Simple hard spherical atomic model (SHSAM) for an *M*/Ag(111)–$\left(\sqrt{3}\times \sqrt{3}\right)R30{}^{\circ}$ surface alloy. (**a**) Positive corrugation. (**b**) Side view of the negative corrugation case in the “monolayer model”. (**c**) Side view of the negative corrugation case in the “bilayer model”. The corrugation parameter *d* is given as the difference between the heights of *M* and Ag atoms: *d = h_M_ – h*_Ag_, where $h_{M}=\sqrt{{\left(r_{\mathrm{Ag}}+r_{M}\right)}^{2}-{\left(\frac{2}{\sqrt{3}}r_{\mathrm{Ag}}\right)}^{2}}$ and $h_{\mathrm{Ag}}=2\sqrt{\frac{2}{3}}r_{\mathrm{Ag}}$.

Figure 3a illustrates the SHSAM in the case for positive corrugation, where the atomic radii of *M* atoms *r_M_* are larger than those of Ag atoms *r*_Ag_. From Figure 3a, we obtained the following equation (Equation (1)) to relate corrugation *d* and atomic radii.

(1) $$d=\sqrt{\left(r_{M}{}^{2}+2r_{\mathrm{Ag}}r_{M}-3r_{\mathrm{Ag}}{}^{2}\right)}\;\;\;\mathrm{for}\;\;\left(r_{M}>r_{\mathrm{Ag}},\;d>0\right)$$

For negative corrugation, we introduced the two models of “monolayer Ag(111) model” and “bilayer Ag(111) model”. Figure 3b,c illustrate the cases for the monolayer and bilayer models, respectively, where the atomic radii of *M* atoms are smaller than those of Ag atoms. From Figure 3b, we obtained the following equation (Equation (2)) for the monolayer model.

(2) $$\;\;\;\;\;d=\;r_{M}-r_{\mathrm{Ag}}\;\;\;\;\mathrm{for}\;\;\;\;\left(r_{M}<r_{\mathrm{Ag}},\;d<0\right)\;\;$$

This corresponds to the situation that hard spheres are on the bottom plane where silver atoms of the monolayer are arranged. From Figure 3c, we obtained the following equation (Equation (3)) for the bilayer model.

(3) $$d=\sqrt{{\left(r_{\mathrm{Ag}}+r_{M}\right)}^{2}-\frac{4}{3}r_{\mathrm{Ag}}{}^{2}}\;-2\sqrt{\frac{2}{3}}\;r_{\mathrm{Ag}}\;\;\;\mathrm{for}\;\;\;\;\left(r_{M}<r_{\mathrm{Ag}},\;d<0\right)$$

For $\;r_{M}>\;r_{\mathrm{Ag}\;}$, $r_{M}=-r_{\mathrm{Ag}\;}+\sqrt{4r_{\mathrm{Ag}}{}^{2}+\;d^{2}}$, for $\;r_{M}<\;r_{\mathrm{Ag}\;}$ with “monolayer model”, $r_{M}=\;d+r_{\mathrm{Ag}}$, and for $r_{M}<\;r_{\mathrm{Ag}\;}$ with “bilayer model”, $r_{M}=\sqrt{d^{2}+4d\sqrt{\frac{2}{3}}\;r_{\mathrm{Ag}}+4r_{\mathrm{Ag}}{}^{2}}-r_{\mathrm{Ag}}$.

This corresponds to the situation that hard spheres are on the underlying Ag(111) layer. A similar equation to (2) can be obtained through expansion of the square root of Equation (3).

The expansion of the square root of Equation (3) to obtained Equation (2). We expand square root for $r_{\mathrm{Ag}}$− $r_{M}$ << 1 in first order and we get $d\approx \sqrt{\frac{3}{2}}\;\left(r_{M}-r_{\mathrm{Ag}}\right)$.

**Figure 4:**
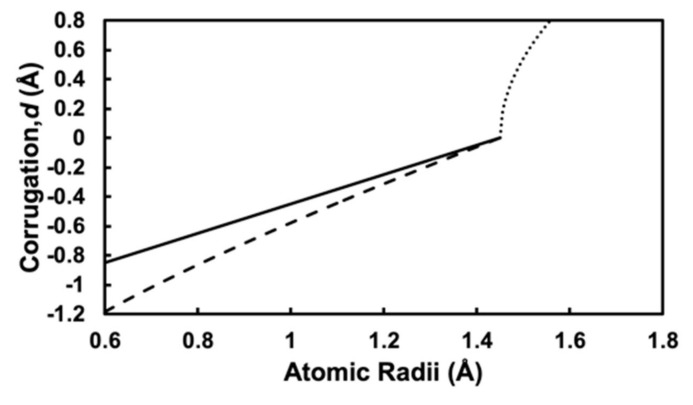
Corrugation parameter *d* calculated with Equations (1)–(3) in the main text, where *r*_Ag_ = 1.45 Å. The dotted line shows positive corrugation given by Equation (1). The solid line and dashed line show negative corrugation given by Equations (2) and (3) with “monolayer model” and “bilayer model”, respectively.

Figure 4 shows the atomic radius *r_M_* dependence of surface corrugations *d* calculated by using Equations (1)–(3), where the atomic radius of silver is evaluated as *r*_Ag_ = 1.45 Å from the SHSAM with an experimental lattice constant *a*_Ag_ = 4.1 Å. The dotted, solid, and dashed lines represent Equations (1)–(3), respectively. For the negative case, the solid line for Equation (2) is absolutely linear, while the dashed line for Equation (3) is almost linear. For the positive case, the dotted line for Equation (1) is nonlinear.

**Figure 5:**
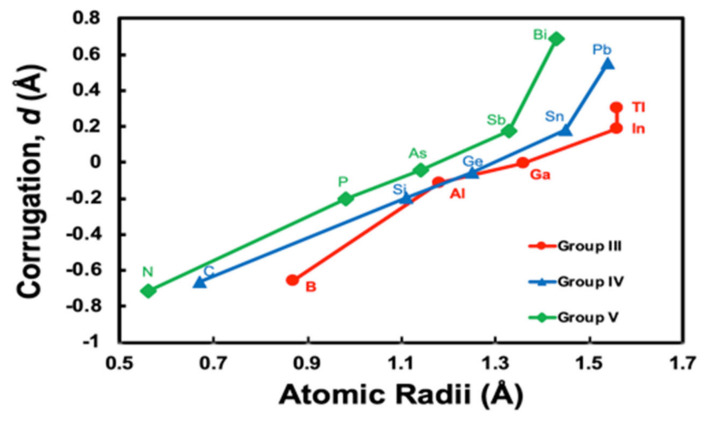
Corrugation parameters *d* in *M*/Ag(111)–$\left(\sqrt{3}\times \sqrt{3}\right)R30{}^{\circ}$ calculated based on the density functional theory (DFT) versus Clementi’s atomic radii of *M* [25]. The lines are a guide to the eye for group III (B, Al, Ga, In, Tl), IV (C, Si, Ge, Sn, Pb), and V (N, P, As, Sb, Bi) atoms.

In Figure 5, we plot the *r_M_* dependence of corrugation parameters with atomic radii of Clementi et al., where the atomic radii are computed with self-consistent field functions based on a minimal basis-set atomic functions for the ground-state atoms [25]. We can clearly see the same tendency; the *r_M_* dependence of corrugation parameters change the gradient at larger atomic radii, as Figure 4 given by the SHSAM, if we plot for the groups III, IV, and V separately. The *r_M_* dependence is classified into three behaviours of Group III, IV, and V, since elements of each group have similar properties based on the same number of valence electrons.

### 3.3. Determination of Surface Alloy Atomic Radii

As we presented in Figure 5, by using Clementi’s atomic radii [25], the *r_M_* dependence of calculated corrugation parameters of surface alloys is qualitatively similar to that from the SHSAM plotted by Equations (1)–(3) in Figure 4. However, from Clementi’s atomic radii and Equation (1) derived from the SHSAM, we cannot explain the positive corrugations, since the atomic radius of an Ag atom is larger than that of any M atom: for example, *r*_Ag_ = 1.65 Å and *r*_Bi_ = 1.43 Å. If we use Clementi’s atomic radii and the SHSAM, all the corrugation parameters should be negative by Equation (2) or Equation (3). Though we also try to explain the surface corrugations using the other type of atomic radii: for example, metallic radii and covalent radii [26] with the SHSAM, it works partly and we cannot reproduce all density functional calculations.

We define a new kind of atomic radii that can generally explain surface corrugations in *M*/Ag(111). The new atomic radii of *r_M_* are obtained from Equations (1)–(3) by entering the DFT corrugation values from our calculations in Table 1, assuming *r*_Ag_ = 1.45 Å as mentioned above. Here, we call the atomic radii determined from corrugations in surface alloys *r_M_* surface alloy atomic radii (SAAR). The calculated SAAR are shown in Table 2. The SAAR are compared with the atomic radii of theoretical calculations by Clementi et al. [25], metallic radii and covalent radii by A. F. Well’s book [26].

**Figure 6:**
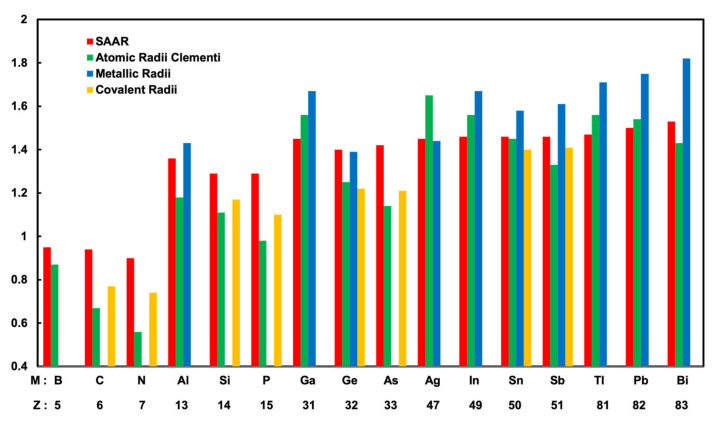
Summary of calculated atomic radii of surface alloy atomic radii (SAAR), Clementi’s atomic radii, metallic radii, and covalent radii, sorted by atomic number Z.

Figure 6 presents the atomic radii of 15 different M atoms and an Ag atom. In Figure 6, the SAAR, Clementi’s atomic radii, metallic radii, and covalent radii are shown in order of the atomic number Z. Clementi’s atomic radii are presented in Table 2 and Figure 6; it is clearly seen that there is a tendency where in the same period, the atomic radius decreases with the increasing atomic number. At this point, for the first (B, C, N) and the second (Al, Si, P) period, the SAAR showed the same tendency as Clementi’s atomic radii. However, in the third and fourth periods, the SAAR did not show the same tendency as Clementi’s atomic radii. In the third period, the SAAR did not have a clear tendency. In the fourth period, the SAAR had the same values of 1.46 Å slightly larger than that of an Ag atom. We found the SAAR also have different tendencies from Clementi’s atomic radii in the fifth period; for Tl, Pb, and Bi, the atomic radii increase with the increasing atomic number. Tl, Pb, and Bi atoms have the SAAR of 1.47, 1.5, and 1.53 Å, respectively. The SAAR for Tl, Pb, and Bi atoms have the same trend as metallic radii, where the radii of Tl, Pb, and Bi atoms are 1.71, 1.75, and 1.82 Å, respectively [26]. We think that the metallic bonding in the cases of M = Tl, Pb, and Bi is important compared to the other periods.

## 4. Conclusions

To understand the origin of the surface corrugations, we performed systematic density functional calculations for silver surface alloys *M*/Ag(111)–$\left(\sqrt{3}\times \sqrt{3}\right)R30{}^{\circ}$, and determined corrugation parameters for *M* of group III, IV, and V elements. The results indicate that the corrugation parameters *d* are related to the atomic number of *M* atoms. Corrugation parameters *d* have tendencies; the value is negative when the atomic number of *M* is smaller than that of Ag, while positive when it is larger.

For further understanding of the tendency of corrugation parameters *d*, we introduced a simple hard spherical atomic model (SHSAM). The atomic radii dependence of corrugation parameters are qualitatively well explained by this model. The surface corrugations can be predicted if we assume the size of atoms determined by atomic radii. However, existing atomic radii cannot be used to predict all surface corrugations with the SHSAM. Then, we inversely determined the surface alloy atomic radii (SAAR) from Equations (1)–(3) derived by the SHSAM.

We discussed corrugation parameters using SAAR and existing atomic radii. SAAR have partly the same tendency as some of existing atomic radii, Clementi’s atomic radii determined by theoretical calculations for isolated atoms [25], covalent atomic radii [26], and metallic atomic radii [26]. The SAAR have the same trend as Clementi’s atomic radii for the first and the second period, while the SAAR have the different trend from Clementi’s but have the same trend as metallic radii for the fifth period. The surface corrugations depend on the nature of chemical bonding, and then, the atomic radii should include such effect. However, existing atomic radii did not include the *M* atom-dependent nature of chemical bonding in the silver surface alloys. Our provided SAAR can be used for simple predictions of surface corrugations without further density functional calculations. It can be used to determine and control corrugations not only in *M*/Ag(111)–$\left(\sqrt{3}\times \sqrt{3}\right)R30{}^{\circ}$ but also in other complex surface alloys applied to spintronics and other fields [27].

## Figures and Tables

**Table 1:** Comparison of corrugation parameters from our calculation and prior works of calculations and experiments, sorted by atomic number Z. *d* denotes the corrugation parameter.

Z	Atom	*d* (Corrugation Parameter) (Å)	Reference (Å)	
			(Theoretical)	(Experimental)
5	B	−0.654	-	
6	C	−0.664	-	
7	N	−0.712	-	
13	Al	−0.115	-	
14	Si	−0.199	-	
15	P	−0.202	-	
31	Ga	−0.002	-	
32	Ge	−0.055	-	0.3 [21]
33	As	−0.040	-	
49	In	0.190	-	
50	Sn	0.184	-	
51	Sb	0.178	-	
81	Tl	0.306	-	
82	Pb	0.556	0.59 [22]	0.42 [22]
83	Bi	0.690	0.69 [23]; 0.61 [9]; 0.65 [24]; 0.8 [14]	0.57 [9]

**Table 2:** Comparison of calculated atomic radii of surface alloy atomic radii and reference.

Atoms	Surface Alloy Atomic radii (Å)	Clementi’s Atomic Radii (Å) [25]	Metallic Radii (Å) [26]	Covalent Radii(Å) [26]
Ag	1.45	1.65	1.44	-
B	0.95	0.87	-	-
C	0.94	0.67	-	0.77
N	0.9	0.56	-	0.74
Al	1.36	1.18	1.43	-
Si	1.29	1.11	-	1.17
P	1.29	0.98	-	1.1
Ga	1.45	1.36	1.53	-
Ge	1.40	1.25	1.39	1.22
As	1.42	1.14	-	1.21
In	1.46	1.56	1.67	-
Sn	1.46	1.45	1.58	1.40
Sb	1.46	1.33	1.61	1.41
Tl	1.47	1.56	1.71	-
Pb	1.5	1.54	1.75	-
Bi	1.53	1.43	1.82	-
